# A multi-wavelength multi-task learning framework for risk-aware fire source classification and smoke density prediction

**DOI:** 10.1038/s41598-026-50282-y

**Published:** 2026-05-04

**Authors:** Yusun Ahn, Hoe-Sung Yang, Kang Bok Lee

**Affiliations:** Defense & Safety Intelligence Research Section, Electronics and Telecommunications Research Institute, 218 Gajeong-ro, Yuseong-gu, Daejeon, 34129 Korea

**Keywords:** Multi-task learning, Multi-wavelength smoke detector, Fire source classification, Smoke density prediction, Process safety, Engineering, Environmental sciences, Mathematics and computing

## Abstract

Traditional smoke detectors suffer from high false alarm rates and lack the ability to differentiate fire sources or quantify smoke density, limiting their effectiveness in diverse indoor environments. While recent studies have explored single-task approaches for fire classification or smoke estimation, these methods typically require separate models, resulting in increased computational overhead. This study proposes a novel multi-task learning (MTL) framework that simultaneously performs fire source classification and smoke density prediction using multi-wavelength optical sensing at 460, 530, 660, and 940 nm. The system was validated under UL 268 standard fire scenarios, including smoldering fires (filter paper and wood), flaming fires (polyurethane foam), and cooking nuisance sources (hamburger patties). Among the evaluated architectures, the CNN–LSTM model achieved the best performance, with a classification accuracy of 97% and a smoke density prediction error of 0.668 MSE. Compared to single-task learning approaches, the MTL framework reduced memory usage by 45% and achieved inference times 82% faster, demonstrating its suitability for real-time edge-based fire detection systems. Ablation studies further revealed that the 940 nm near-infrared wavelength provides discriminative features for distinguishing fire smoke from representative nuisance aerosols. Overall, the proposed approach enables reliable early detection with reduced false alarms in indoor environments.

## Introduction

Fires pose a significant global threat, resulting in substantial loss of life and extensive property damage each year. A considerable proportion of these losses is attributed to inadequate response during the early stages of fire development. Consequently, smoke detectors that can rapidly identify smoke and provide timely alarms have become essential safety devices for protecting occupants and assets in indoor environments^1,2^. By detecting smoke at an early stage, smoke detectors are vital for mitigating fire-related risks in indoor environments^3–5^.

In indoor environments, however, reliable smoke detection remains challenging due to the frequent presence of non-fire aerosols such as dust, cooking fumes, and exhaust gases. These aerosols often exhibit scattering and absorption characteristics similar to those of fire smoke, making accurate discrimination between fire and non-fire events difficult^6–8^. Furthermore, variations in indoor activities lead to significant temporal changes in aerosol composition and concentration, which contribute to persistent false alarms. From a process and building safety perspective, false alarms represent more than operational inconvenience, as repeated nuisance alarms can induce alarm fatigue, delayed response, and reduced trust in safety systems.

In parallel, analog smoke detectors have been increasingly adopted to mitigate false alarms, particularly in South Korea and other regions^9^. Unlike conventional detectors, which only determine fire presence, analog detectors measure smoke density and light transmittance, reporting values in terms of obscuration (OBS) [%/m]. This approach enables more accurate detection of transient events and provides actionable warnings based on smoke concentration levels^10^. Moreover, smoke density data not only aids in detection but also provides essential information for evacuation planning, false alarm reduction, and fire prevention research, as highlighted by several studies^11–14^.

Despite these advantages, the potential of smoke density information has not been fully exploited in intelligent fire detection systems. Most prior studies treat smoke source identification and smoke density estimation as independent tasks, leading to increased computational complexity and limited suitability for real-time, resource-constrained safety systems.

To overcome these limitations, this study proposes a multi-task learning (MTL)-based smoke detection model that simultaneously performs smoke source classification and smoke density prediction^15^. By sharing representations between related tasks, multi-task learning improves learning efficiency and generalization performance^16,17^. Integrating qualitative event identification with quantitative hazard estimation enables a transition from conventional alarm-based detection toward risk-aware fire safety systems.

**Fig. 1:**
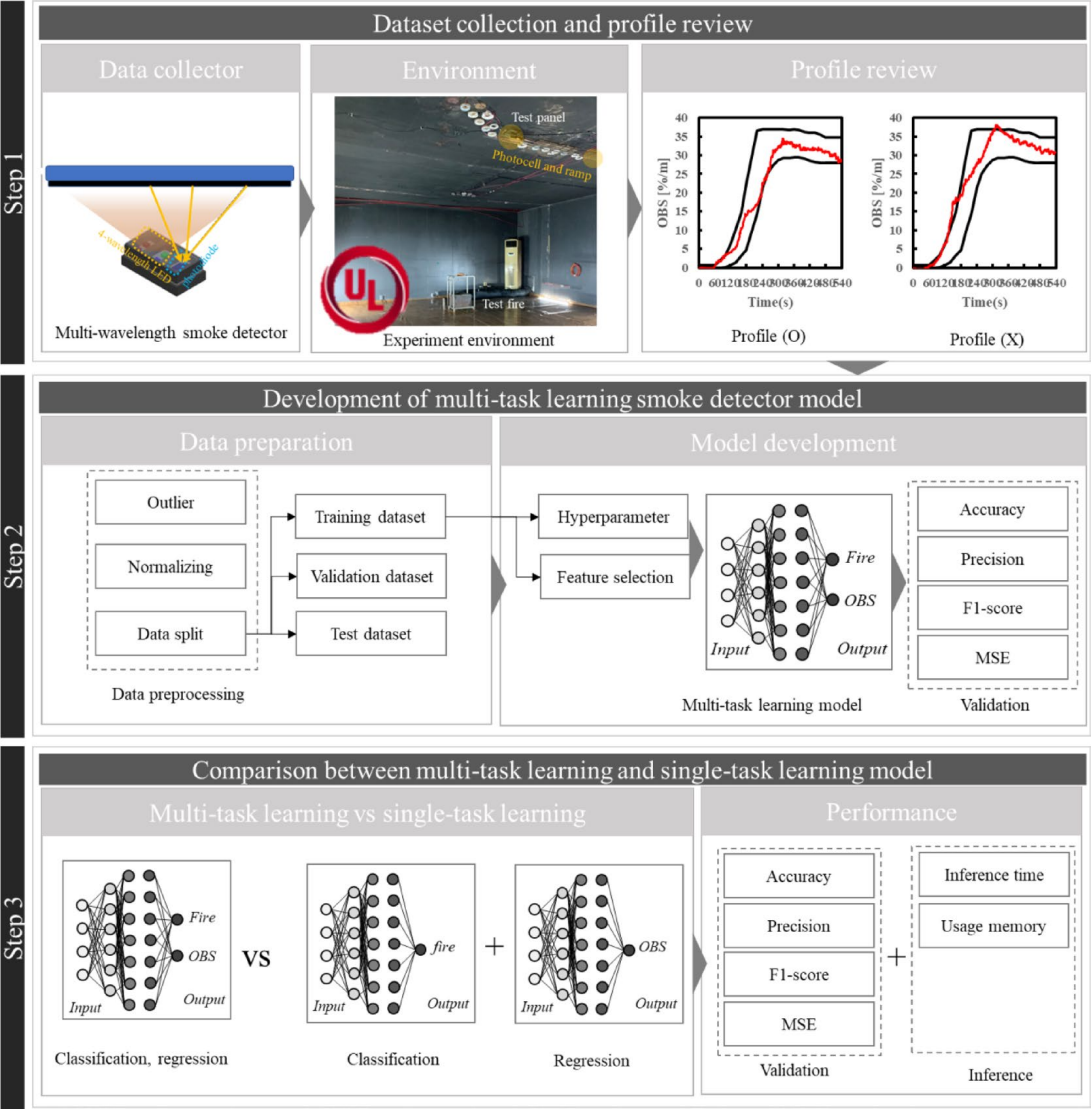
Research framework of the proposed multi-task learning-based smoke detection system.

The proposed framework is developed and validated through three key steps, as illustrated in Fig. 1. First, experimental data are collected under real-world fire and nuisance scenarios in accordance with the Underwriters Laboratories (UL) 268 standard^18^, ensuring practical relevance and reliability. Second, a multi-task learning model is constructed using multi-wavelength optical sensing at 460, 530, 660, and 940 nm to simultaneously classify smoke sources and predict smoke density. Finally, the performance of the proposed model is experimentally evaluated and compared with conventional single-task learning approaches.

This study aims to advance smoke detection technology for indoor environments by demonstrating robust discrimination between fire smoke and representative nuisance aerosols such as cooking fumes and water vapor under UL 268-compliant conditions. Moreover, the proposed approach provides a foundation for future risk-aware fire safety systems that leverage smoke density information for enhanced decision-making in process and building safety applications.

## Related work

Fire detection research has evolved from simple threshold-based systems to sophisticated multi-sensor and learning-based frameworks^1,19–22^. Two major research streams have emerged to address the limitations of conventional single-wavelength detectors: multi-modal sensor fusion and multi-wavelength optical sensing. This section reviews both streams and identifies the research gap addressed by the present study.

Conventional detectors and their limitations. Traditional smoke detectors rely on a single infrared wavelength (~ 900 nm) to detect scattered light from smoke particles. While widely deployed, these systems cannot distinguish between fire smoke and non-fire aerosols such as dust, cooking fumes, and water vapor^6,7^, leading to persistent false alarms. This limitation has motivated the exploration of more discriminative detection approaches^8,20,23–25^.

To improve detection reliability, recent studies have integrated heterogeneous sensor data. Liu et al.^26^ combined temperature, smoke concentration, and CO sensors to detect early-stage fires. Baek et al.^27^ developed a multi-modal system integrating temperature, humidity, CO, CO_2_, O_2_, dust, and smoke sensors, achieving reliable early-stage detection through a weighted dynamic time warping algorithm. However, this approach is limited to binary fire/non-fire classification and does not address smoke density estimation or source identification, leaving a gap in simultaneous multi-task hazard assessment. Chen et al.^28^ demonstrated that multi-source fusion significantly reduces false alarm rates. Collectively, these studies confirm that sensor diversity improves detection reliability. However, increased sensor complexity raises system cost and limits deployment in resource-constrained environments.

An alternative strategy exploits wavelength-dependent scattering properties of aerosol particles. Li et al.^29^ developed a dual-wavelength detector that estimated aerosol particle size from scattering intensity ratios to reduce false alarms. Deng et al.^30^ measured aerosol surface area and volume using dual-wavelength sensing for accurate particle size differentiation. Wegrzynski et al.^23^ extended this to five wavelengths for robust particle size analysis across diverse fire types. Özyurt O^24,25^ further demonstrated deep learning-based aerosol classification using multi-wavelength scattering. While these studies demonstrate strong spectral discrimination capability, they remain limited to single-task classification and do not address simultaneous hazard quantification.

Deep learning models have been increasingly applied to fire and smoke detection^31,32^. For instance, CNN-LSTM-based architectures applied to multi-wavelength smoke data have demonstrated competitive classification performance^33,34^. However, most existing approaches treat fire source classification and smoke density estimation as independent tasks, requiring separate models and incurring additional computational overhead.

**Fig. 2:**
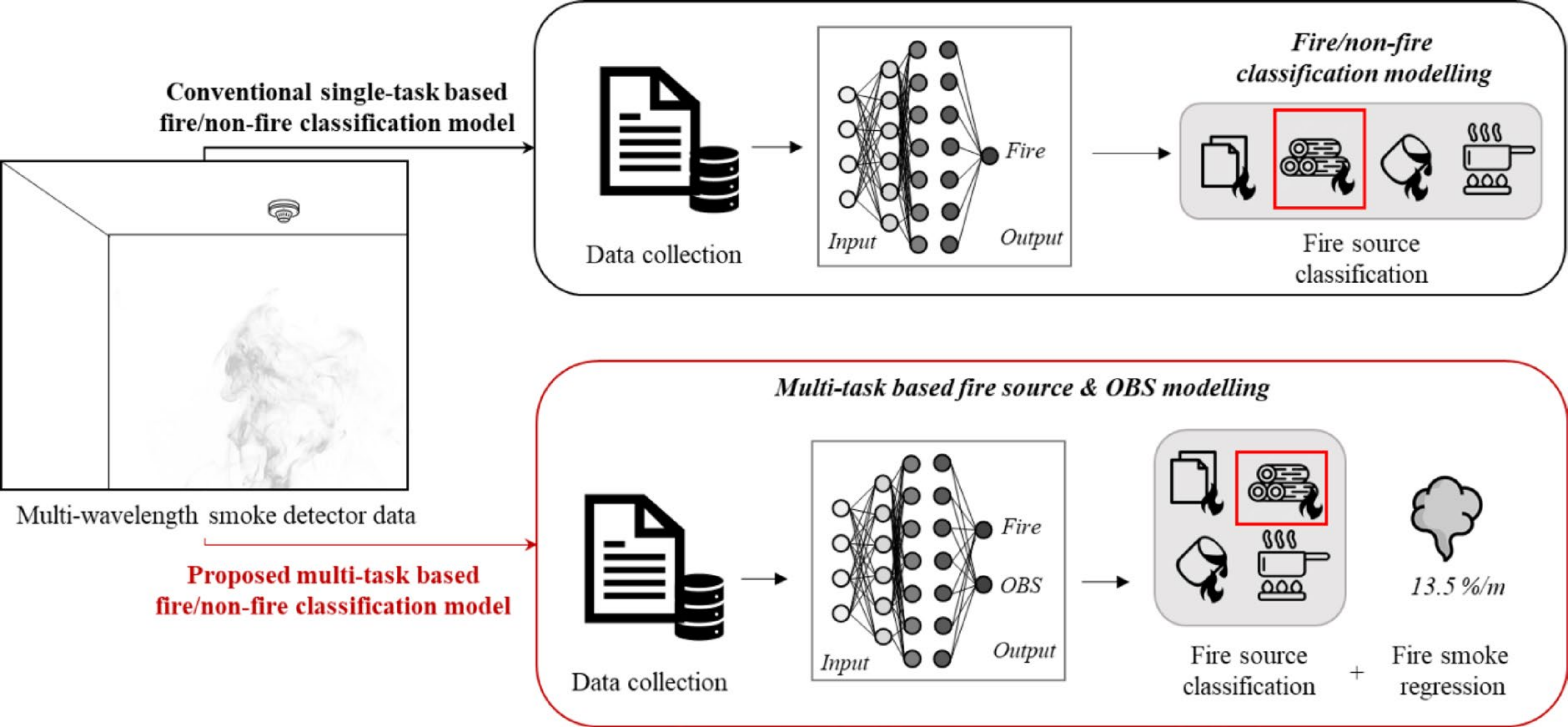
Comparison of conventional single-task approaches and the proposed MTL framework highlighting the research gap.

As shown in Table 1, existing studies have not yet proposed a unified framework that simultaneously performs fire source classification and smoke density prediction using multi-wavelength sensing. The present study addresses this gap by introducing a multi-task learning (MTL) framework that shares representations across both tasks, enabling simultaneous classification and regression while reducing computational cost. Notably, unlike most prior studies conducted under laboratory conditions, this study employs real-world fire scenarios compliant with the UL 268 standard^18^, enhancing practical reliability. Figure 2 illustrates the key differences between the present study and previous approaches.

**Table 1:** Summary of various previous studies related to the smoke detectors.

Author	Wavelengths [nm]/sensors	Method	Experimental setup		Prediction		
			Full scale	Standards	Fire/non-fire	Fire source	OBS
Liu et al.^26^	Temperature, smoke concentration, and CO	Multi-modal/Single-task	X	–	O	–	–
Baek et al.^27^	Temperature, humidity, CO, CO_2_, O_2_, dust, and smoke sensors	Multi-modal/Single-task	O	–	O	–	–
Chen et al.^28^	CO, temperature, smoke, and humidity	Multi-modal/Single-task	X	–	O	–	–
Dong et al.^35^	Particle size platform	Single-task	X	–	O	O	–
Zheng et al.^8^	405, 850	Single-task	X	–	O	O	–
Özyurt O^25^	405, 980	Single-task	X	EN-54-7	O	O	–
Özyurt O^24^	450, 980	Single-task	X	–	O	O	–
Li et al.^29^	470, 940, LED	Single-task	X	EN-54	O	O	–
Han et al.^34^	460, 533, 664, 947	Single-task	X	–	O	O	–
Węgrzyński et al.^23^	450, 520, 658, 830, 980	Single-task	O	EN-54-7			–
This study	460, 530, 660, 940	Multi-task	O	UL 268	O	O	O

## Materials and methods

### Multi-wavelength smoke detector

**Fig. 3:**
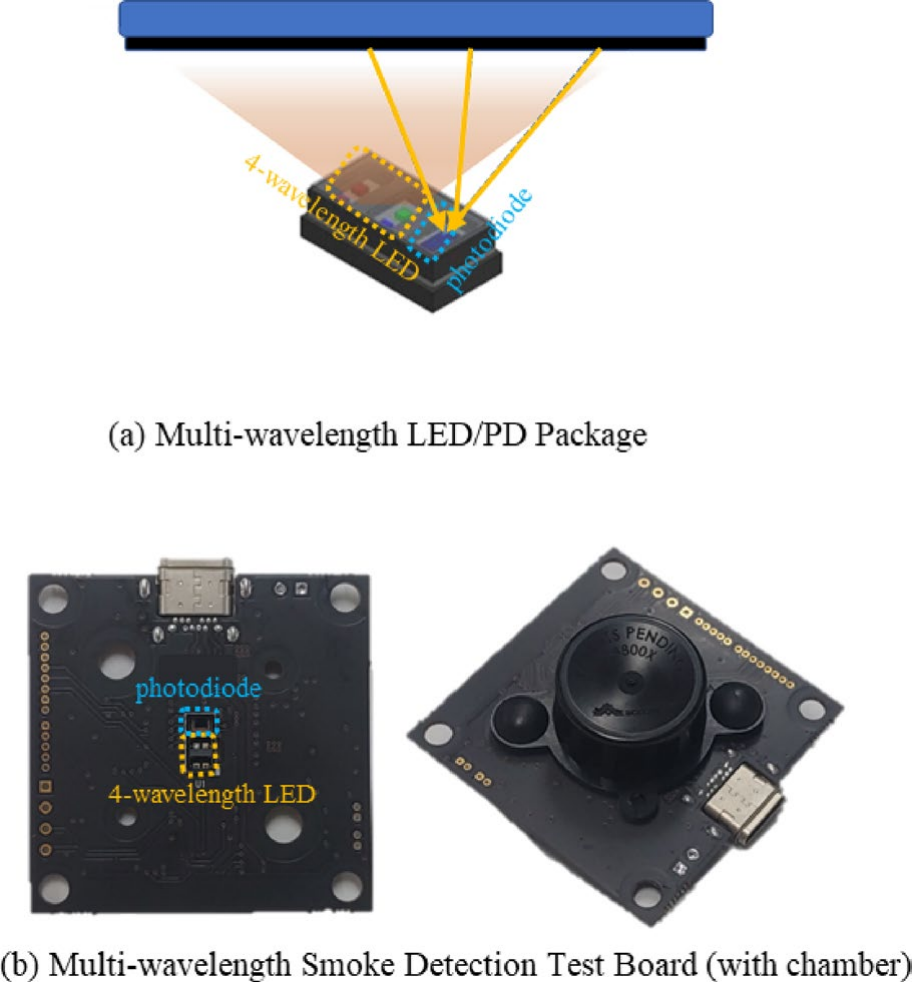
Multi-wavelength smoke detector employing four LEDs at 460, 530, 660, and 940 nm.

Conventional smoke detectors commonly operate using an infrared light source in the 900 nm range, detecting scattered light from smoke particles entering the detector. However, this method merely determines the presence of particles without distinguishing between fire and non-fire sources. To address this limitation, this study hypothesized that the scattering properties of aerosol particles vary with the wavelength of the light source. Accordingly, this study developed a multi-wavelength smoke detector, as illustrated in Fig. 3, to measure scattering properties across multiple wavelengths.

The multi-wavelength smoke detector constructed in this study employs four types of LEDs with wavelengths of 460 nm, 530 nm, 660 nm, and 940 nm, paired with photodiodes in a packaged configuration. The LEDs and photodiodes are controlled using a time-division multiplexing method, enabling the measurement of scattering properties at each wavelength.

The use of multiple wavelengths in smoke detection plays a crucial role in identifying the source of smoke. The scattering characteristics of smoke vary depending on the wavelength and the type of fire source. Since the particle size and density of smoke differ according to the material, the scattering behavior changes across different wavelengths. This is because the interaction of light with smoke particles is proportional to the particle size relative to the wavelength^36,37^.

When the particle size is comparable to or larger than the wavelength, Mie scattering^38^ dominates, while Rayleigh scattering^39^ occurs primarily when the particle size is smaller than the wavelength. At shorter wavelengths, Rayleigh scattering is predominant, as the smoke particles are typically smaller or comparable to the wavelength. This is particularly effective for detecting fine smoke particles produced during incomplete combustion. At intermediate wavelengths, Mie scattering becomes more prominent, as the particle size often exceeds the wavelength. This effect is especially pronounced for solid materials undergoing combustion. At longer wavelengths, Mie scattering continues to dominate and is useful for detecting water vapor or the moisture content in smoke. By leveraging these characteristics, experiments were conducted using a multi-wavelength smoke detector to classify smoke sources.

### Multi-task learning model

Multi-task learning has been successfully applied in various domains, including medical imaging^40^, natural language processing^41^, and skin lesion analysis^42^. However, its application to multi-wavelength-based smoke detection remains underexplored, especially in safety–critical systems where simultaneous event classification and hazard quantification are essential. This study seeks to address this gap.

Before selecting the final model, this study evaluated several architectures, including the Convolutional Neural Network-Long Short-Term Memory (CNN-LSTM) model, which demonstrated high performance in single-task learning in previous research by the authors^33^. While prior studies primarily focused on single-task learning, this study extends the CNN-LSTM architecture to accommodate the multi-task learning paradigm. Additionally, this study compared it with various additional models to identify the most suitable architecture for multi-task learning.

Four representative deep learning architectures were evaluated to identify the most suitable framework for multi-task smoke detection, considering both performance and computational efficiency.

1. CNN-LSTM^43,44^ combines a convolutional feature extractor with a Long Short-Term Memory (LSTM) network. The CNN layers capture local patterns and spatial features from the multi-wavelength input, while the LSTM layers model sequential dependencies across time steps. This architecture is well-suited for time-series sensor data where both local feature extraction and temporal context are critical.
2. Transformer^45,46^ relies on a self-attention mechanism to model global dependencies across all time steps simultaneously, without relying on recurrence. This enables the capture of long-range temporal relationships in sequential data. However, its computational cost scales quadratically with sequence length, which may limit efficiency in resource-constrained deployments.
3. CNN-GRU^47,48^ replaces the LSTM with a Gated Recurrent Unit (GRU), which uses a simplified gating structure (update and reset gates) compared to LSTM’s three-gate design. This reduces the number of parameters and computational overhead while retaining the ability to model temporal dependencies, making it a strong candidate for memory-efficient edge deployment.
4. CNN^49,50^ serves as a baseline architecture that applies convolutional layers solely for spatial feature extraction, without any recurrent or attention-based temporal modeling. Including this model allows assessment of the contribution of temporal modeling to overall multi-task performance.

**Fig. 4:**
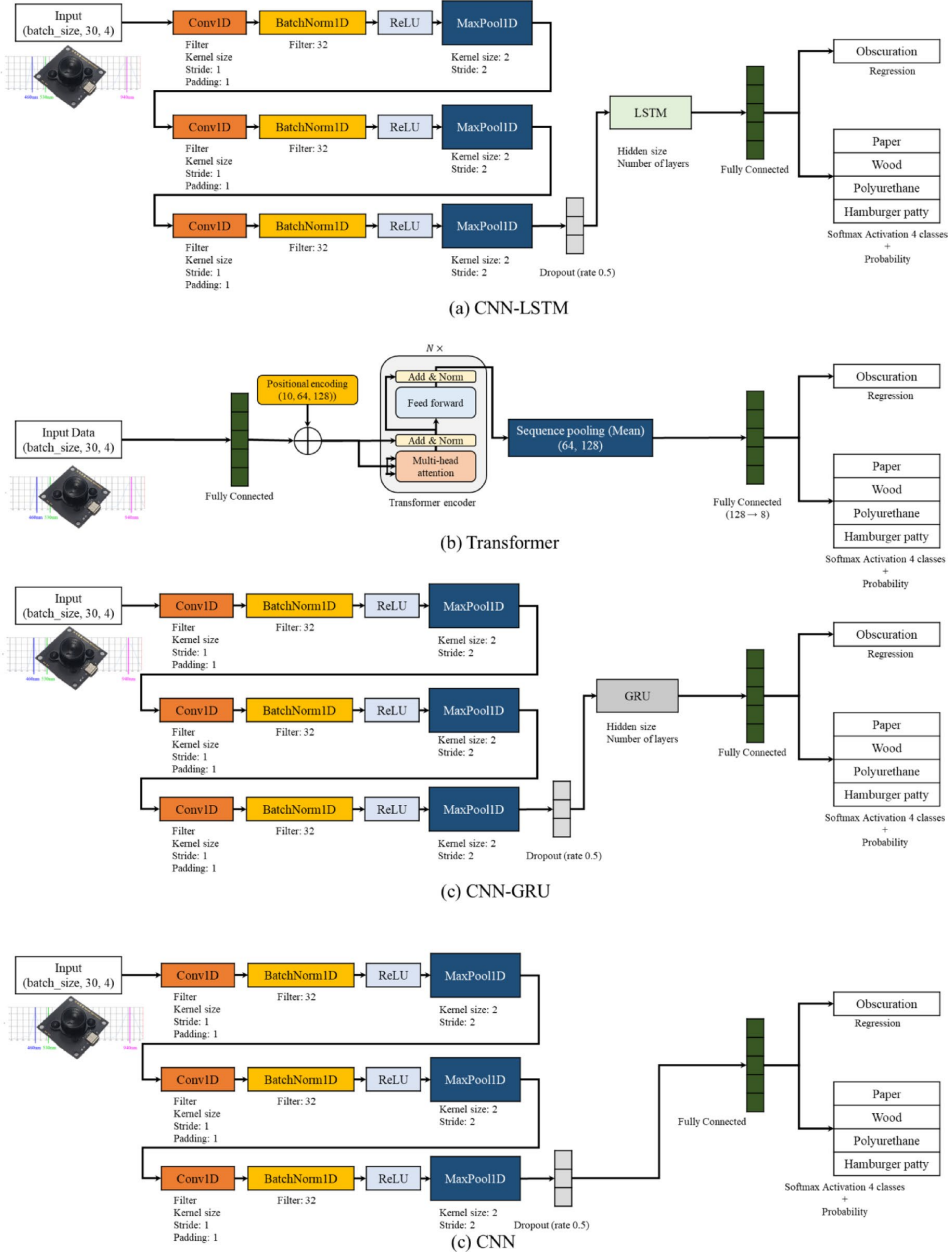
Architectures of the four deep learning models evaluated: CNN-LSTM, Transformer, CNN-GRU, and CNN.

For model training, a system running Windows 11 with an NVIDIA GeForce RTX 4060 Ti GPU, Python 3.11.5, CUDA 12.1, and PyTorch 2.2.1 was used. Training was conducted for up to a maximum of 3,000 epochs to ensure sufficient convergence with a time step of 30 and data sampled at 10 s intervals. To monitor overfitting, validation loss was tracked during training. The validation loss reached a minimum at epoch 277, after which it increased, indicating the onset of overfitting. Accordingly, the model weights at epoch 277 were selected as the final model via best-model checkpointing. Regularization was further supported by Dropout (rate = 0.3) and Batch Normalization layers incorporated in the model architecture, as well as the implicit regularization effect of the multi-task learning framework, in which shared parameters must simultaneously satisfy both classification and regression objectives. The architectures and performances of the models are illustrated in Fig. 4.

### Data preprocessing

#### Multi-wavelength data normalization

The input data for the multi-wavelength smoke detector developed in this study consist of scattering intensities measured at four different wavelengths, resulting in four distinct features. Since the scattering intensities vary depending on the wavelength, both in magnitude and range, normalization was performed to enable direct comparison between the features.

**Fig. 5:**
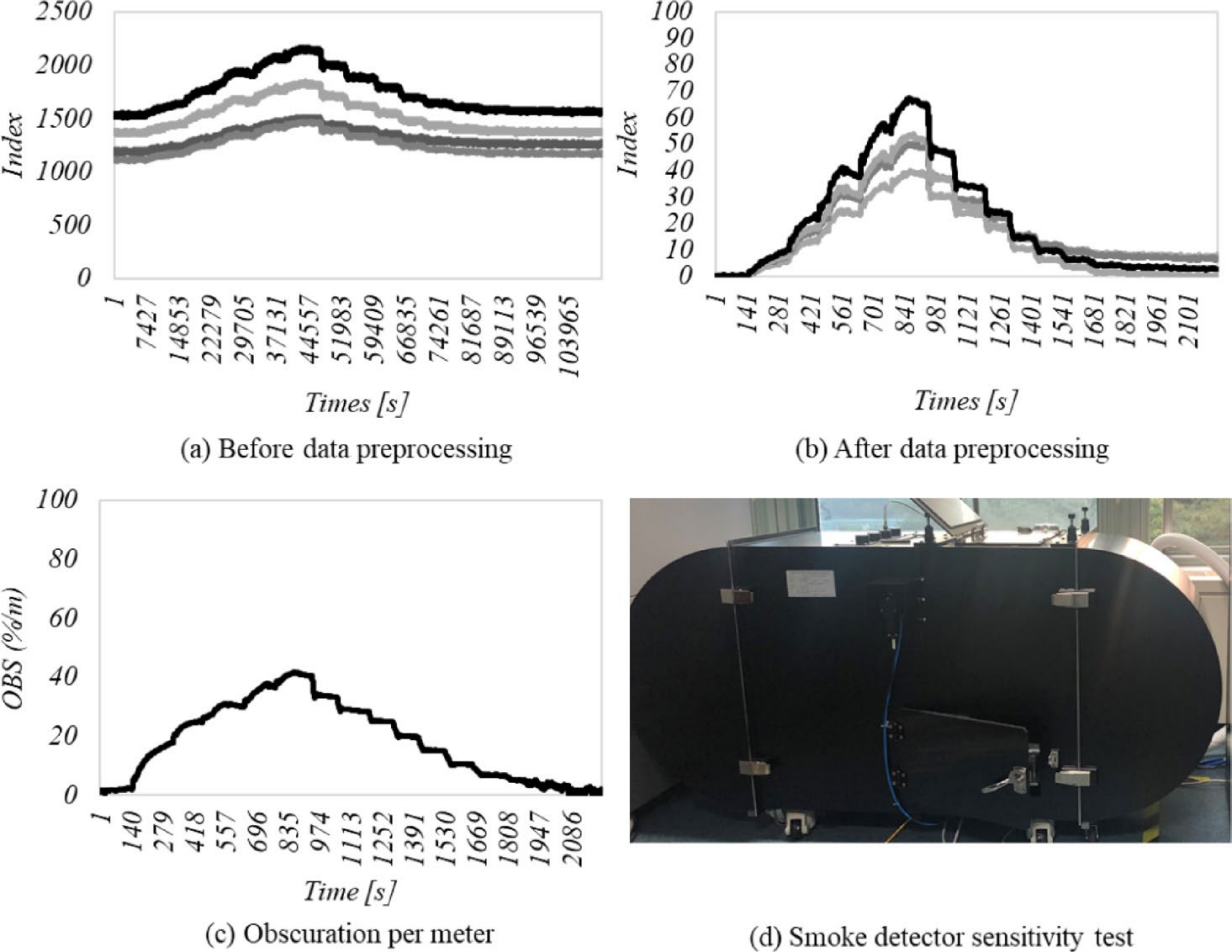
Multi-wavelength scattering data: (**a**) raw signals, (**b**) normalized signals, (**c**) OBS measurements, and (**d**) sensitivity tester.

The multi-wavelength smoke detector collects 50 data points per second. To minimize the impact of outliers, the median value of these 50 data points was selected for processing. The collected data were normalized using Eq. (1), where ${x}_{mean, 30s}$ represents the mean value of the data collected during the first 30 s after the detector is activated. The normalized data processed using Eq. (1), are visualized in Fig. 5. Figure 5a shows the raw multi-wavelength data before preprocessing, Fig. 5b illustrates the normalized data after preprocessing, and Fig. 5c presents the corresponding OBS measurements obtained using a precision photocell and lamp.

1 $$x^{\prime} = \left\{ {\left( {\frac{x}{{x_{mean, 30s} }}} \right)^{{\frac{1}{0.4}}} - 1} \right\} \times 100$$

The exponent term in Eq. (1) was determined based on the physical configuration of the smoke detector sensitivity tester used in this study. Specifically, the optical path length between the light source and the photocell in the tester was 0.4 m. Therefore, the normalization formulation was designed to be consistent with the white light obscuration model described in Eq. (2), where the path length is explicitly considered.

The OBS measurements were performed using a smoke detector sensitivity tester, as shown in Fig. 5d. This tester, commonly used in Korea for developing and verifying smoke detectors, generates smoke by placing filter paper on a hotplate. The smoke concentration is adjusted by controlling the amount of filter paper, while the built-in photocell and lamp measure light transmission to provide precise OBS values.

Equation (1) was designed to closely align the preprocessed data in Fig. 5b with the precise OBS measurements shown in Fig. 5c. It was inspired by Eq. (2), which is based on the white light obscuration system used by Underwriters Laboratories^51^ . The light signal measured by the LED can be expressed in OBS as follows:

2 $${\mathrm{OBS}} = \left( {1 - \left( {\frac{I}{{I_{0} }}} \right)^{1/d} } \right) \times 100$$

Here, $\mathrm{I}$ represents the measured light intensity, ${I}_{0}$ is the initial light intensity, and $\mathrm{d}$ denotes the path length.

#### Anomaly detection point

In this study, the data collected from the multi-wavelength smoke detector consist of four features, which necessitates a clear and conservative definition of the anomaly detection starting point. The anomaly detection point is defined not as the ignition moment of a fire source, but as the time at which smoke reaches the detector and the measured signals become distinguishable from background conditions through model inference.

The anomaly detection threshold was carefully determined to balance early detection and false alarm mitigation. According to Korean standards, conventional smoke detectors typically trigger fire alarms at an OBS level of 15 %/m. In contrast, this study defines the anomaly detection starting point at an early-stage OBS range of 2–3 %/m, where multi-wavelength scattering patterns begin to deviate from normal background behavior while remaining well below the regulatory alarm threshold.

Within the 2–3 %/m OBS range, scattering intensity patterns still vary depending on the fire source. Therefore, a rule-based refinement criterion was introduced to ensure stable anomaly detection across different fire scenarios. This criterion was empirically derived based on repeated observations of normalized multi-wavelength scattering behavior across all experimental conditions and is expressed in Equation (3).

3 $$if \left( {x_{940 nm}^{^{\prime}2} - x_{460 nm}^{^{\prime}2} } \right)\left\langle {0.01 \vee x_{940 nm}{\prime} } \right\rangle 1.02 \vee x_{460 nm}{\prime} > 0.995$$

Here, ${x}^{^{\prime}}$ denotes the normalized scattering intensity at each wavelength. The threshold values used in Eq. (3) were empirically determined through repeated observation of normalized scattering patterns across all fire scenarios. These values were selected in a conservative manner to ensure that anomaly detection consistently occurs within the target OBS range of 2–3%/m, while minimizing premature triggering caused by background noise or transient non-fire aerosols. This rule-based criterion is employed as a preprocessing step to identify reliable early-stage smoke events prior to multi-task model inference, rather than as a final alarm decision mechanism.

#### Feature selection for input data

The quality and quantity of features in training data directly influence a model’s performance and computational efficiency^52,53^. Therefore, appropriate feature selection, including the addition and removal of features, is essential for building accurate and efficient models. In this study, data from the multi-wavelength detector were used to experimentally analyze 15 feature combinations by adding or removing specific features from the four available wavelengths.

#### Hyperparameter selection

Hyperparameter optimization was performed using the GridSearch algorithm^54^ , a widely used method for improving machine learning model performance. GridSearch exhaustively searches all predefined hyperparameter combinations to identify the best-performing configuration. This study used the parameter module provided by scikit-learn to optimize the hyperparameters listed in Table 2.

**Table 2:** Hyperparameter search space for each model.

Hyperparameter	Model			
	CNN-LSTM	Transformer	CNN-GRU	CNN
learning_rate	0.001, 0.0001	0.001, 0.0001	0.001, 0.0001	0.001, 0.0001
hidden_size	32, 64, 128	–	32, 64, 128	–
num_layers	1, 2, 3	2, 4, 6	1, 2, 3	–
batch_size	16, 32	16, 32	16, 32	16, 32
Num_head	–	2, 4, 8	–	–

Each hyperparameter was selected based on its impact on training and model performance. *Learning rate* controls the speed of parameter updates; too high a value causes divergence, while too low a value results in slow convergence, with values of 0.001 and 0.0001 explored. *Hidden size* affects model capacity and computational cost, with larger sizes increasing both. *Number of layers* determines model depth; deeper models can capture complex patterns but may suffer from gradient vanishing or overfitting. *Batch size* specifies the number of samples per training step and affects memory usage and training stability. *Number of heads* refers to the parallel attention heads in the Transformer’s Multi-Head Attention mechanism.

### Experimental setup

#### UL268 test condition

UL 268^18^ provides standards for the requirements of smoke detectors used in commercial and residential fire alarm systems. These standards focus on ensuring that smoke detectors are capable of accurately detecting fires and issuing timely alerts. The standard outlines specifications for the design, performance, testing methods, and certification processes of smoke detectors.

Experiments were conducted based on the “fire test,” which evaluates fire detection performance, and the “cooking nuisance smoke test,” which assesses false alarm prevention, as defined by UL 268. The standard specifies two key components for testing: test room conditions and profiles.

##### Test room condition

**Fig. 6:**
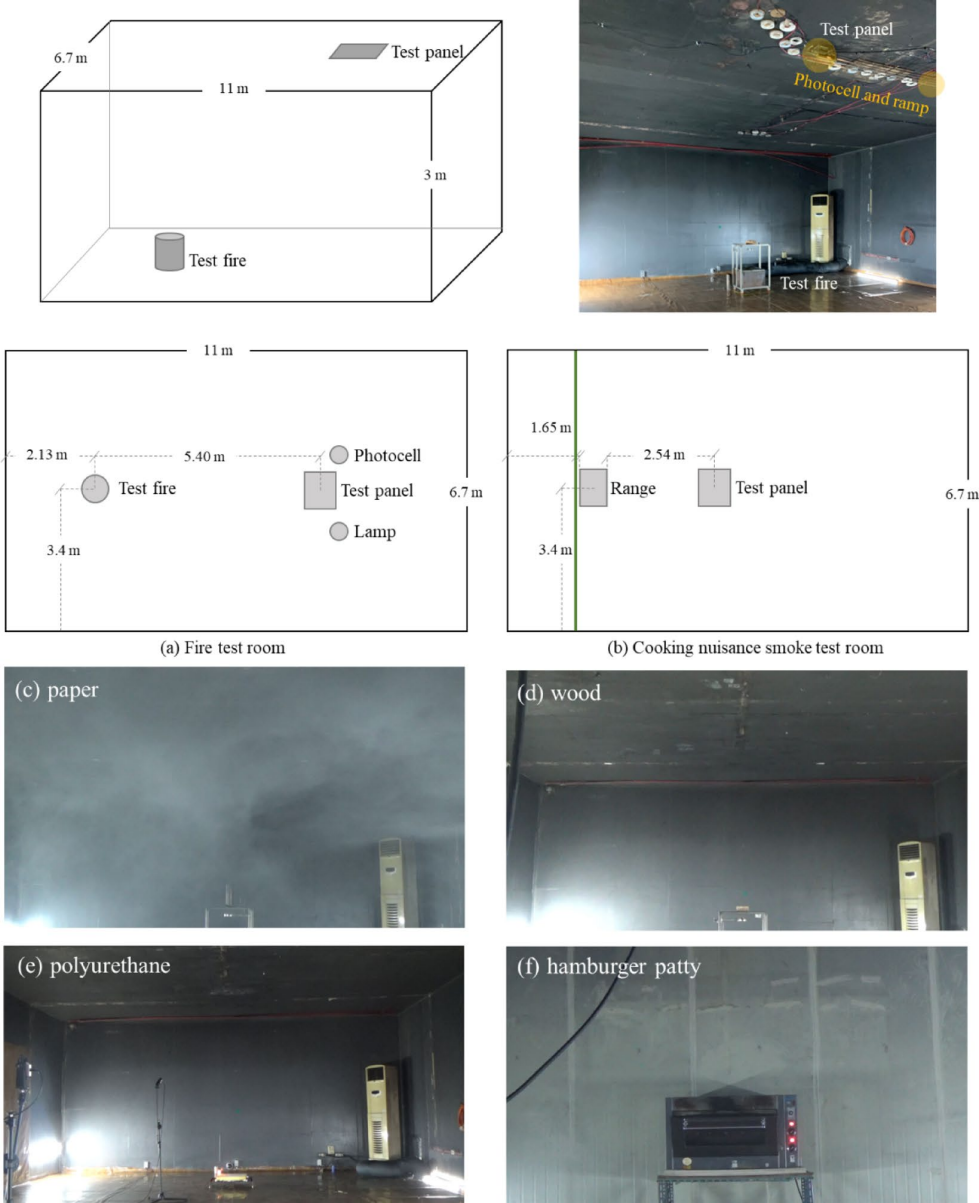
UL 268 test room configurations and combustible materials used in fire and nuisance smoke tests.

The fire test room must meet the dimensions of 11 m (length), 6.7 m (width), and 3 m (height), as shown in Fig. 6. The combustibles for the “fire test” include paper, wood, and polyurethane, as illustrated in Fig. 6c–e. For the “cooking nuisance smoke test,” the combustible is hamburger patty, as shown in Fig. 6e.

For the “fire test,” the setup must satisfy the conditions shown in Fig. 6a, with combustibles placed 2.13 m away from the wall. For the “cooking nuisance smoke test,” the setup must meet the conditions shown in Fig. 6b, with combustibles positioned 1.65 m away from the nearest wall and 0.05 m from the range’s side wall. Additionally, all combustibles must be placed on a support 0.9 m above the floor. All experiments were conducted in full compliance with these conditions.

##### Profile

UL 268 specifies smoke profiles that must be matched during testing. While fuel amounts are suggested, they can be adjusted to meet the required profiles. Table 3 presents the profiles outlined in UL 268. For paper and wood combustibles, the smoke detector must detect fire within 4 min of ignition. For polyurethane, the smoke detector must activate before the smoke reaches 15.47%/m OBS. For hamburger patties, the detector must not activate if the smoke remains below 4.84%/m OBS. Although UL 268 provides various profile conditions (e.g., OBS vs. Time, CO vs. OBS), this study focused solely on OBS vs. Time performance.

**Table 3:** Summary of profiles and combustibles.

Profile	Description
	Profile (i) Flame breakthrough is to occur at between 1 and 3 min (ii) The first principle peak is to occur at between 1 and 3 min (iii) Smoke is to peak at 64.4 and 78.1%/m obscuration (iv) There is to be between 20 and 40 s of 12.56%/m (v) The secondary peak is not to exceed 36.7%/m
	Combustibles Shredded newsprint: 6–10 mm (width), 25.4–102 mm (length) 42.6 g (weight)
	Profile (i) Smoke buildup is to begin at between 80 and 120 s (ii) There is to be at least 60 s of 12.56%/m or higher obscuration (iii) Maximum obscuration is not to exceed 45.8%/m obscuration (iv) Flame breakthrough is to occur at between 150 and 190 s (v) The test shall be 4 min after ignition
	Combustibles A wood brand formed of three layers of kiln dried fir strips: 19.1 mm square in cross section, 152 mm long with six strips, thick 19 mm, diameter 102 mm before cooking
	Profile The requirements outlined in polyurethane profile All detectors shall produce an alarm signal at or before an obscuration limit of 15.47%/m
	Combustibles A pure polypropylene oxide polyol, polyether-based flexible polyurethane foam 368 by 432 by 76 mm
	Profile The requirements outlined in hamburger patty profile All detectors shall not produce an alarm signal at the obscuration level of 4.84%/m
	Combustibles Mixture of 75 percent lean beef and 25 percent suet by weight ground together at least twice

To validate the profiles provided by UL 268, a photocell and lamp system was installed to measure OBS. This system continuously monitored changes in smoke concentration by forming an optical path using the lamp as a stable light source and the photocell to detect scattering and absorption caused by smoke particles. The measured values were converted into OBS, serving as a quantitative indicator of smoke density.

**Fig. 7:**
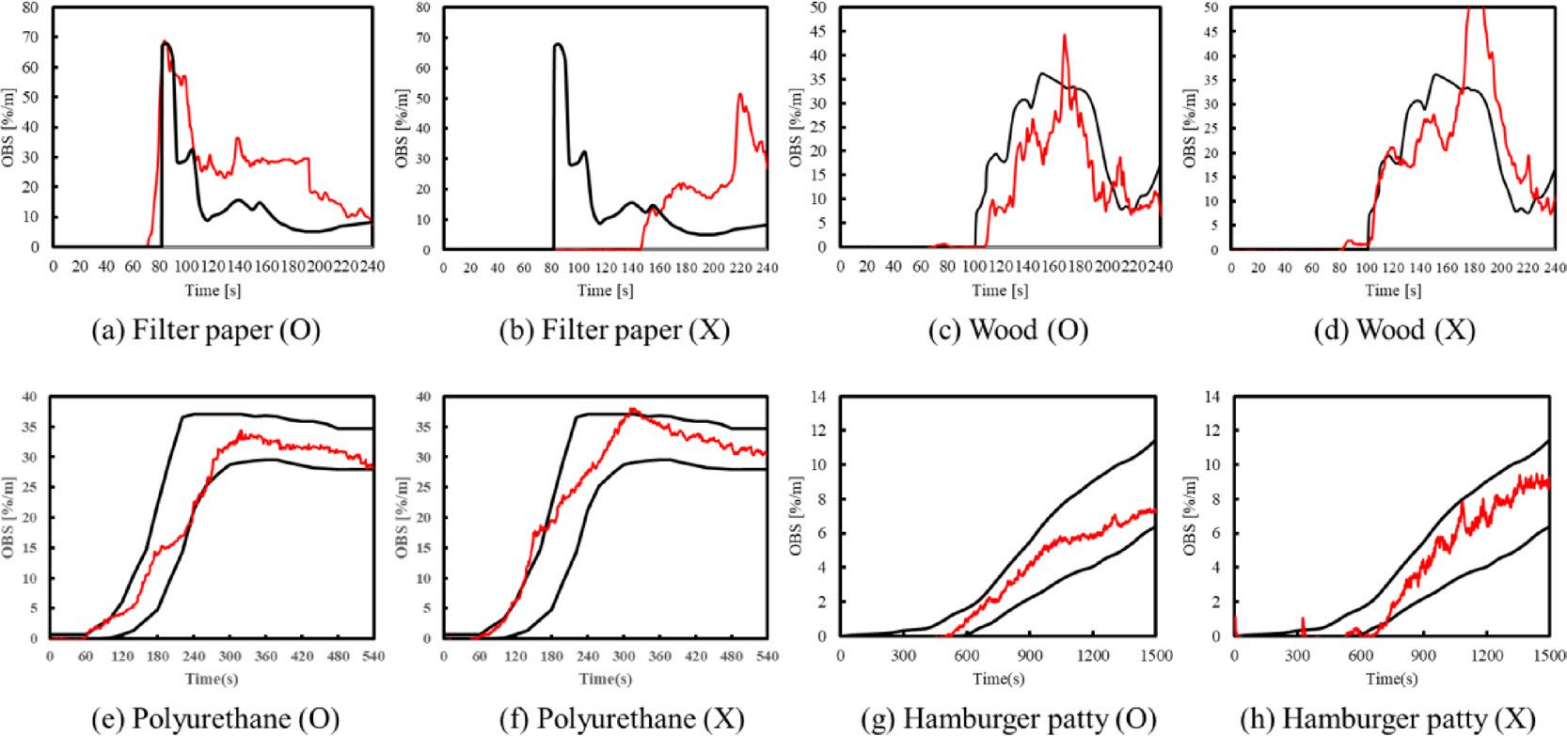
Measured OBS profiles (red) versus UL 268 standard profiles (black). (O): compliant; (X): non-compliant.

As smoke density increased, the OBS values captured these dynamic changes, and a time-dependent profile was generated. Figure 7 shows the results, where the black line represents the UL 268 profile, and the red line represents the OBS data measured in this study. Profiles matching UL 268 standards were marked as (O), while those that did not match were marked as (X). Although profiles marked with (X) do not strictly satisfy all UL 268 criteria, they were retained to capture realistic variations in smoke generation observed in real-world environments. These data were not used for regulatory compliance evaluation but were included to enhance the robustness and generalization capability of the proposed model.

Profiles marked with (O) indicate data that meet UL 268 standards and are considered valid for further analysis. In contrast, profiles marked with (X) represent conditions that do not strictly adhere to UL profile specifications but reflect real-world variability in smoke generation encountered in operational environments. Smoke generation in actual fire scenarios is influenced by numerous factors—including fuel moisture content, ambient conditions, ventilation, and ignition dynamics—which may deviate from the controlled conditions defined by UL 268. By including these realistic data points in the training dataset, this study aims to enhance model robustness and practical applicability across diverse fire detection scenarios, including those encountered where environmental conditions are less controlled than laboratory settings.

### Training dataset

In the UL 268 tests, data were collected 16 times for each fire source. Table 4 summarizes the datasets used for training, validation, and testing, categorized by the presence or absence of the UL 268 profile. Out of the 16 tests, 14 were used for training, 1 for validation, and 2 for testing. Among these, three datasets per fire source met the UL 268 profile, while the remaining 13 did not.

**Table 4:** Number of experimental datasets and profile success.

	Filter paper		Wood		Polyurethane		Hamburger patty	
	Profile O	Profile X	Profile O	Profile X	Profile O	Profile X	Profile O	Profile X
Train	2	11	2	11	2	11	2	11
Validation	–	1	–	1	–	1	–	1
Test	1	1	1	1	1	1	1	1

For the training dataset, two experiments that satisfied the profile were used alongside non-profile-compliant data. The test dataset included one profile-compliant dataset and one non-compliant dataset. Each test involved the use of seven multi-wavelength sensors installed per experiment.

Although the number of experiments per fire source is relatively limited, the effective sample size for model training is substantially larger. Each experiment generates continuous time-series data sampled at 10-s intervals over the full test duration, and the sliding window approach (time step = 30) produces a large number of training observations per experiment. As summarized in Table 4, the total training dataset comprises 2,296 labeled observations across all fire source categories, providing a sufficiently large dataset for training the deep learning models employed in this study.

To mitigate the risk of overfitting associated with limited experiment counts, several strategies were adopted. First, non-profile-compliant datasets were deliberately included in training to expose the model to realistic variability in smoke generation patterns, thereby improving generalization beyond controlled UL 268 conditions. Second, the inclusion of seven spatially distributed sensors per experiment effectively multiplied the diversity of training observations by capturing sensor-position-dependent scattering variations. Third, hyperparameter optimization was performed using GridSearch with a held-out validation set to prevent overfitting during model selection.

Regarding temporal correlation, the train and test sets were split at the experiment level rather than the time-step level, ensuring that no temporal leakage occurred between training and test data. Each test experiment was conducted independently from the training experiments, thereby preserving the integrity and reliability of the model evaluation.

### Evaluation criteria

The evaluation criteria for classification and regression models differ, as the objectives of these tasks are distinct. Classification models focus on accurately predicting predefined categories, while regression models aim to minimize the error between predicted and actual values. In this study, the performance of the classification model for fire source detection and the regression model for smoke density prediction were evaluated using appropriate metrics.

The metrics for classification include precision (Eq. (4)), recall (Eq. (5), f1-score (Eq. (6)), and Accuracy (Eq. (7)). Precision measures the proportion of true positives among all samples predicted as true. Recall quantifies the proportion of true positives among all actual positive samples. F1-Score is the harmonic mean of Precision and Recall. Accuracy indicates the proportion of correctly predicted samples among all samples:

4 $$Precision = \frac{TP}{{TP + FP}}$$

5 $$Recall = \frac{TP}{{TP + FN}}$$

6 $$F1 - score = 2 \times \frac{Precison \times Recall}{{Precision + Recall}}$$

7 $$Accuracy = \frac{TP + TN}{{TP + TN + FP + FN}}$$

Here, TP (True Positive): Correctly predicted as true when it is true. FP (False Positive): Incorrectly predicted as true when it is false. FN (False Negative): Incorrectly predicted as false when it is true. TN (True Negative): Correctly predicted as false when it is false.

For regression, the Mean Squared Error (MSE) was selected as the evaluation metric (8). MSE quantifies the average squared difference between actual values (${y}_{i}$) and predicted values (${\widetilde{y}}_{i}$), n represents the total number of data points. Beyond model performance, this study also evaluated the inference time and memory usage of each model to assess their practicality in edge device environments.

8 $$MSE = \frac{1}{n}\mathop \sum \limits_{i = 1}^{n} \left( {y_{i} - \tilde{y}_{i} } \right)^{2}$$

## Results

The multi-task learning framework developed in this study was designed to improve smoke detection accuracy and smoke density prediction by utilizing combinations of multi-wavelength data. The optimized hyperparameters obtained through GridSearch, as described in Table 2, are summarized in Table 5. Based on these configurations, the models were trained over 3,000 epochs.

**Table 5:** Optimized hyperparameters.

Hyperparameters	Model			
	CNN-LSTM	Transformer	CNN-GRU	CNN
learning_rate	0.001	0.001	0.0001	0.001
hidden_size	32	–	128	–
num_layers	2	6	2	–
batch_size	32	32	16	32
Num_head	–	8	–	–

### Model performance evaluation and visualization

#### Model comparison results

The performance of the trained models was evaluated based on two tasks: fire source classification and smoke density prediction. As shown in Table 6, CNN-LSTM, CNN-GRU, and CNN models all achieved an accuracy of 0.97 in fire source classification, demonstrating superior performance. In contrast, the Transformer model exhibited a lower accuracy of 0.92. For smoke density prediction, the CNN-LSTM model recorded the lowest MSE of 0.668, validating the suitability of the multi-task learning approach. The Transformer model had the highest MSE of 0.822, which aligns with prior studies suggesting that while Transformer models excel at extracting semantic correlations, they may face limitations in modeling temporal relations^55^.

**Table 6:** Results of multi-task learning model.

	Accuracy	MSE	Peak memory used	Average inference time
CNN-LSTM	0.97	0.668	129.91 MB	0.004 s
Transformer	0.92	0.822	763.31 MB	0.062 s
CNN-GRU	0.97	0.740	113.61 MB	0.004 s
CNN	0.97	0.706	98.30 MB	0.029 s

The Transformer model’s performance may be improved by optimizing hyperparameters such as positional encoding or by integrating task-specific architectural modifications. Future work could explore Transformer-based models tailored for multi-task learning in smoke detection.

In terms of efficiency, the Transformer model consumed the most memory (763.31 MB), due to the computational complexity of its self-attention mechanism. This poses limitations in memory-constrained, real-time environments. In contrast, the CNN-LSTM (129.91 MB), CNN-GRU (113.61 MB), and CNN (98.30 MB) models demonstrated significantly lower memory usage, making them more efficient.

The Transformer model also had the slowest average inference time at 0.062 s, which can be attributed to its computational demands. On the other hand, the CNN-LSTM and CNN-GRU models achieved the fastest inference times (0.004 s), while the CNN model recorded a slightly slower time (0.029 s), still suitable for real-time applications.

In conclusion, the CNN-LSTM model achieved the best overall performance, with an accuracy of 0.97 and an MSE of 0.668, demonstrating its robust generalization capabilities across various fire sources and smoke density conditions. While the Transformer model’s computational requirements limit its practicality, the CNN-GRU and CNN models offer strong efficiency and remain viable alternatives for real-time deployment.

#### Performance of CNN-LSTM model

The results of models other than the CNN-LSTM are provided in the Appendix. Classification results are presented in Tables 10, 11, and 12, and regression results are illustrated in Figs. 12, 13, and 14. The performance of the CNN-LSTM model is summarized in Table 7, highlighting its effectiveness in fire source classification and smoke density prediction. Notably, the model achieved the highest performance for “Normal,” “Polyurethane,” and “Hamburger Patty” categories, with relatively lower F1-scores for “Paper” and “Wood.” Despite these variations, the model demonstrated stable performance with macro and weighted averages both achieving 0.97.

**Table 7:** Classification results of CNN-LSTM model (Accuracy: 0.97).

Confusion matrix				
	Precision	Recall	F1-score	Support
Normal	0.97	0.98	0.98	585
Paper	0.97	0.95	0.96	334
Wood	0.96	0.95	0.96	457
Polyurethane	1.00	0.97	0.98	441
Hamburger patty	0.96	1.00	0.98	479
Accuracy			0.97	2296
Macro avg	0.97	0.97	0.97	2296
Weighted avg	0.97	0.97	0.97	2296

**Fig. 8:**
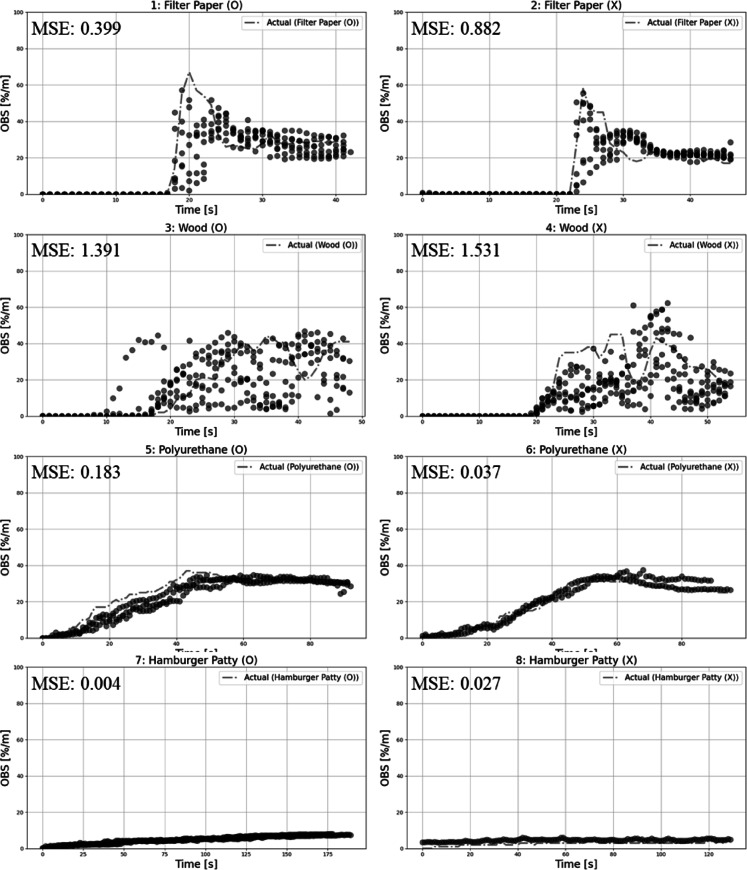
Regression results of CNN-LSTM regression model (MSE: 0.668). Gray dashed lines: actual OBS; black dots: sensor predictions.

Regression results comparing predicted and actual smoke densities are visualized in Fig. 8. The x-axis represents time, while the y-axis represents smoke density. Markers (O) and (X) indicate whether the data complies with the UL 268 profile. The gray dashed line represents actual OBS values obtained through photocell and lamp measurements, while black dots indicate predictions from the seven sensors. The black dots indicate predictions obtained from seven smoke detectors installed simultaneously at different locations within the UL 268 test room.

For “Filter Paper” and “Wood,” sharp changes in smoke density resulted in relatively lower accuracy (Filter Paper: MSE 0.399 (O), 0.882 (X); Wood: MSE 1.391 (O), 1.531 (X)). In contrast, “Polyurethane” and “Hamburger Patty” exhibited smoother density changes, leading to higher accuracy (Polyurethane: MSE 0.183 (O), 0.037 (X); Hamburger Patty: MSE 0.004 (O), 0.027 (X)).

Overall, the CNN-LSTM model demonstrated strong performance in both fire source classification and smoke density prediction. The model provided reliable results for UL 268-compliant (O) data and showed practical applicability for non-compliant (X) data, which reflect real-world variability. These results confirm that the CNN-LSTM model offers a balanced combination of performance and efficiency, making it an optimal choice for multi-task learning in smoke detection.

#### Multi-task learning vs. single-task learning

**Fig. 9:**
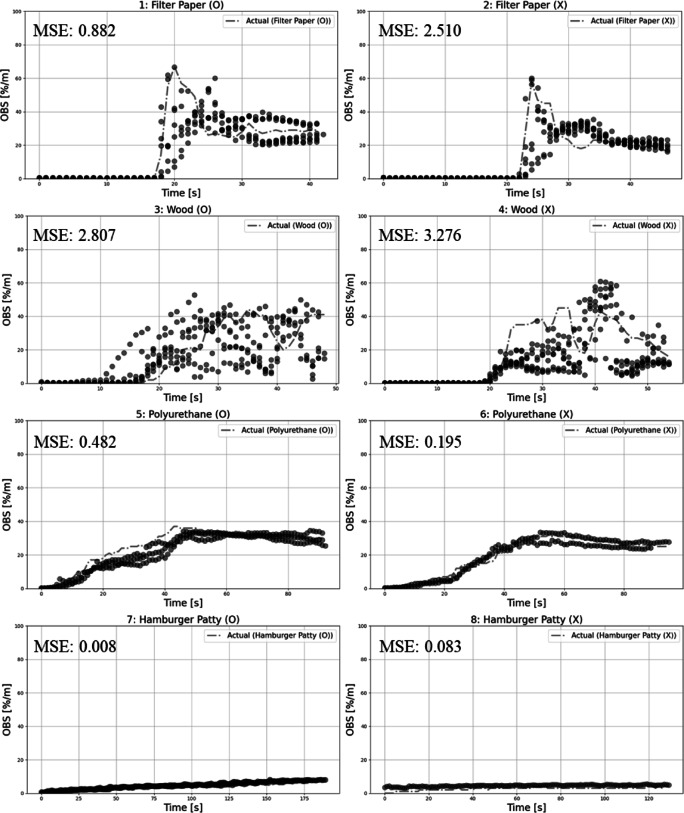
Smoke density regression results of the single-task learning model (MSE: 1.858).

The performance of the Multi-task Learning (MTL) model was also compared with that of Single-task Learning (STL) models. Each STL model independently performed either classification or regression tasks. The classification results are summarized in Table 8, and the regression results are presented in Fig. 9.

**Table 8:** Classification results of single-task learning (Accuracy: 0.96).

Confusion matrix				
	Precision	Recall	F1-score	Support
Normal	0.99	0.97	0.98	590
Paper	0.96	0.88	0.92	332
Wood	0.92	0.98	0.95	455
Polyurethane	0.99	0.95	0.97	441
Hamburger patty	0.95	1.00	0.97	478
Accuracy			0.96	2296
Macro avg	0.96	0.96	0.96	2296
Weighted avg	0.96	0.96	0.96	2296

For fire source classification, MTL and STL models achieved accuracies of 0.97 and 0.96, respectively. For smoke density prediction, the MTL model outperformed the STL model, recording an MSE of 0.668 versus 1.858 (approximately 2.8 times lower). This difference indicates that task-specific training in STL limits regression performance due to the absence of shared representations.

The regression performance of the STL model, illustrated in Fig. 9, shows a notable difference compared to the MTL model. The STL model’s higher MSE of 1.858 underscores its limitation in predicting smoke density when tasks are performed independently.

**Fig. 10:**
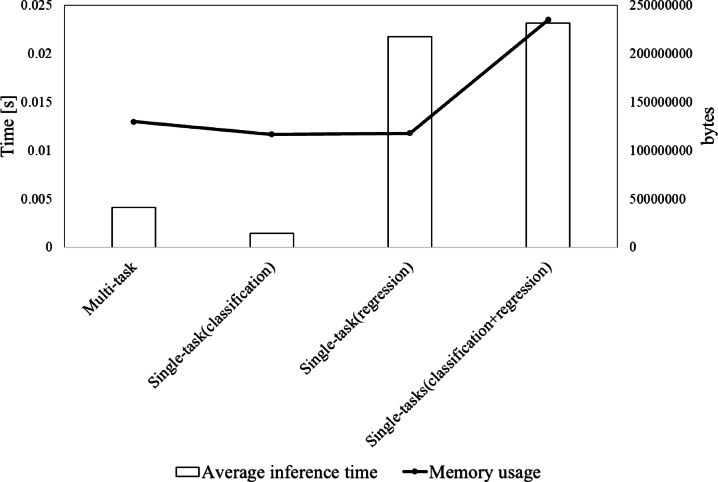
Comparison of peak memory usage and average inference time between MTL and STL models.

Figure 10 visually compares the inference time and peak memory usage between MTL and STL models. The MTL model recorded a peak memory usage of 129.96 MB, representing a ~ 45% reduction compared to the combined memory usage of the STL models (234.87 MB).

In terms of inference time, the MTL model achieved an average of 0.004142 s per inference. This is significantly faster than the combined average inference time of the STL models, which recorded 0.02318 s (Classification: 0.001427 s; Regression: 0.021753 s). Conversely, the STL models required separate memory allocations for each task, with peak memory usage of 116.81 MB for classification and 118.06 MB for regression, resulting in a combined usage of 234.87 MB, much higher than the MTL model.

The MTL model demonstrated clear advantages over STL models by reducing memory usage and performing both tasks simultaneously. This dual-task capability is especially beneficial in resource-constrained edge environments, making MTL a practical choice for real-time detection systems. Additionally, the MTL model achieved a lower MSE for smoke density prediction, benefiting from the shared information between classification and regression tasks.

### Effect of wavelength combinations on model performance

The performance of 15 feature combinations derived from four wavelengths was evaluated in terms of classification accuracy, F1-score, and mean squared error (MSE). The results for each feature combination are summarized in Table 9. The experimental findings reveal clear performance variations depending on the selected wavelength combination, with the inclusion of all four wavelengths yielding the best overall results. When all four wavelengths were used, the model achieved an accuracy of 0.97, an average F1-score of 0.97, and an MSE of 0.668, representing the highest performance among all evaluated configurations. This indicates that multi-wavelength input enhances both classification reliability and smoke density prediction accuracy.

**Table 9:** Performance comparison of feature combinations based on wavelength selection.

Feature 1 case		Feature 2 case		Feature 3 case		(base) Feature 4 case	
Accuracy, F1-score(avg)	MSE	Accuracy, F1-score(avg)	MSE	Accuracy, F1-score(avg)	MSE	Accuracy, F1-score(avg)	MSE
$x_{460}^{{}}$		$x_{460}^{{}}$, $x_{530}^{{}}$		$x_{460}^{{}}$, $x_{530}^{{}}$,$x_{660}^{{}}$		$x_{460}^{{}}$, $x_{530}^{{}}$, $x_{660}^{{}}$,$x_{940}^{{}}$	
0.78, 0.74	1.213	0.88, 0.86	0.899	0.94, 0.93	0.842	0.97, 0.97	0.668
$x_{530}^{{}}$		$x_{460}^{{}}$, $x_{660}^{{}}$		$x_{460}^{{}}$, $x_{530}^{{}}$,$x_{940}^{{}}$			
0.75, 0.70	1.443	0.87, 0.84	0.792	0.92, 0.90	0.920		
$x_{660}^{{}}$		$x_{460}^{{}}$, $x_{940}^{{}}$		$x_{460}^{{}}$, $x_{660}^{{}}$,$x_{940}^{{}}$			
0.85, 0.82	1.005	0.91, 0.90	0.876	0.96, 0.96	0.774		
$x_{940}^{{}}$		$x_{530}^{{}}$, $x_{660}^{{}}$		$x_{530}^{{}}$, $x_{660}^{{}}$,$x_{940}^{{}}$			
0.70, 0.67	1.227	0.81, 0.79	0.990	0.94, 0.93	0.931		
		$x_{530}^{{}}$, $x_{940}^{{}}$					
		0.85, 0.81	1.045				
		$x_{660}^{{}}$, $x_{940}^{{}}$					
		0.92, 0.92	0.848				

In contrast, single-wavelength inputs exhibited substantially degraded performance. For example, using only the 460 nm wavelength yielded the lowest accuracy (0.78), F1-score (0.74), and a high MSE (1.213), indicating that reliance on a single spectral channel is insufficient for robust smoke characterization. Notably, feature combinations that included longer wavelengths, particularly 660 nm and 940 nm, consistently demonstrated higher accuracy and more balanced F1-scores, with reduced discrepancies between classification and regression performance. These results provide quantitative evidence for the discriminative contribution of the 940 nm wavelength, consistent with the wavelength-dependent scattering characteristics of smoke particles in the near-infrared (NIR) range. Formal statistical significance testing was not performed in this study, and this is acknowledged as a limitation.

The importance of the 940 nm wavelength is quantitatively supported by the ablation study results presented in Table 9. Feature combinations including the 940 nm channel consistently achieved higher classification accuracy (mean: 0.899 vs. 0.856) and lower MSE (mean: 0.901 vs. 1.006) compared to combinations without it. Notably, the highest performance was achieved only when all four wavelengths, including 940 nm, were used (accuracy: 0.97, MSE: 0.668).

Overall, the ablation results confirm that multi-wavelength input is essential for achieving robust and reliable smoke detection performance, justifying the design choice of employing a four-wavelength sensing configuration in the proposed multi-task learning framework.

## Discussion

The results in Section “Effect of wavelength combinations on model performance” reveal that accuracy improves as the feature combinations include higher wavelengths, particularly when $x_{940}^{{}}$ is present. These findings indicate that the 940 nm wavelength provides distinct characteristics for fire source classification, especially in environments where non-fire aerosols such as dust, welding fumes, and exhaust gases are prevalent. While other wavelengths also contribute to performance, the proportional relationship among all four wavelengths enhances the system’s overall discrimination capability.

**Fig. 11:**
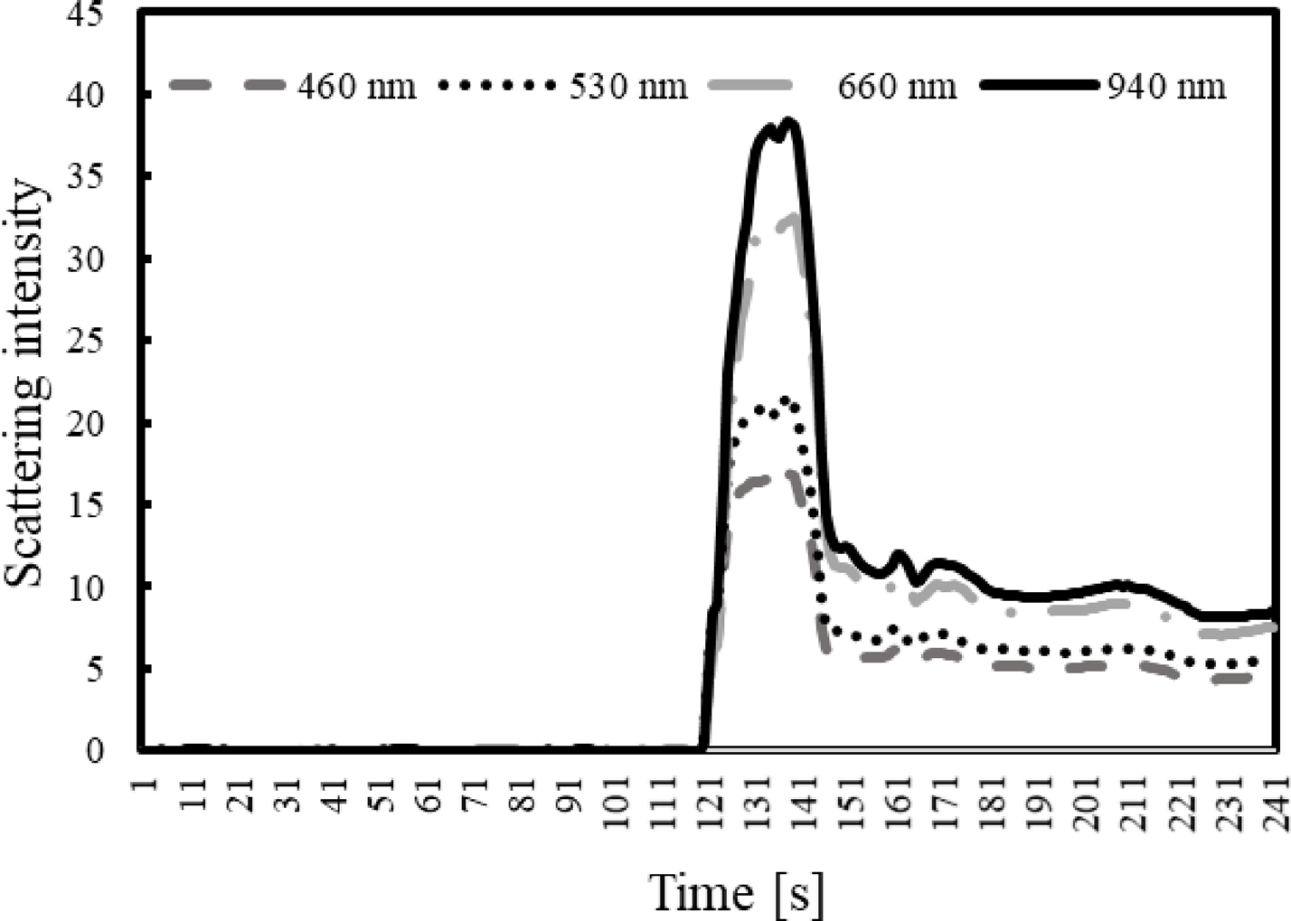
Multi-wavelength scattering intensity during the filter paper fire test, showing distinctly higher values at 940 nm.

**Fig. 12:**
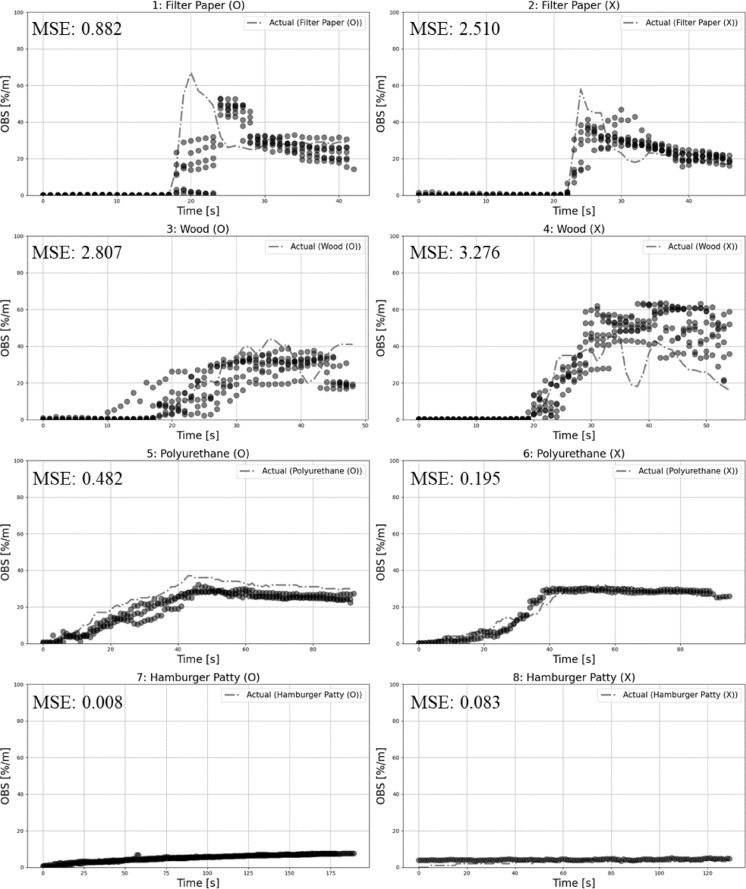
Regression results of Transformer model.

**Fig. 13:**
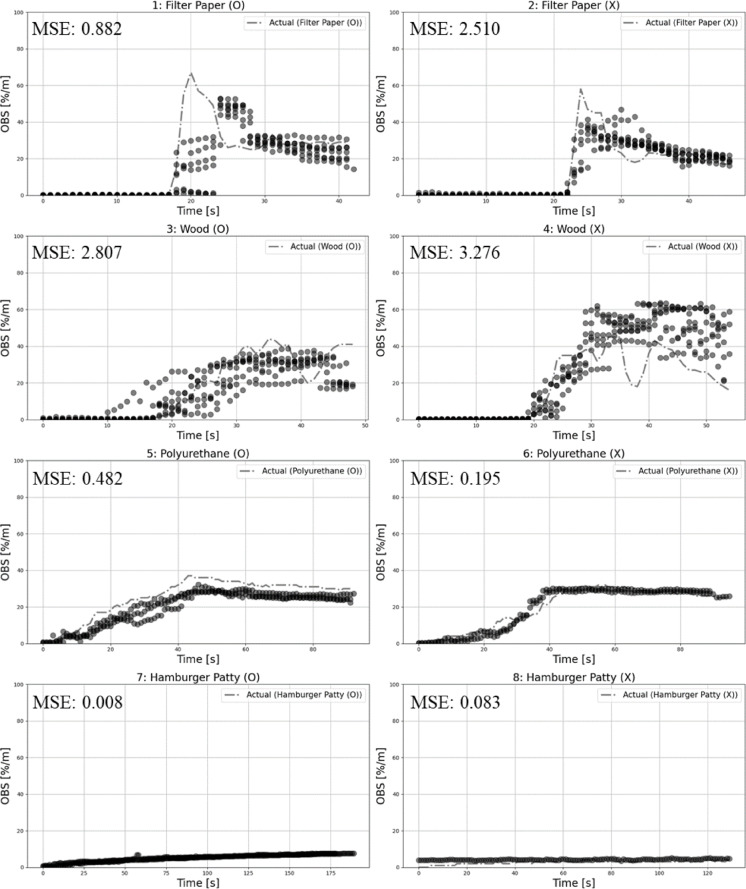
Regression results of CNN-GRU model.

**Fig. 14:**
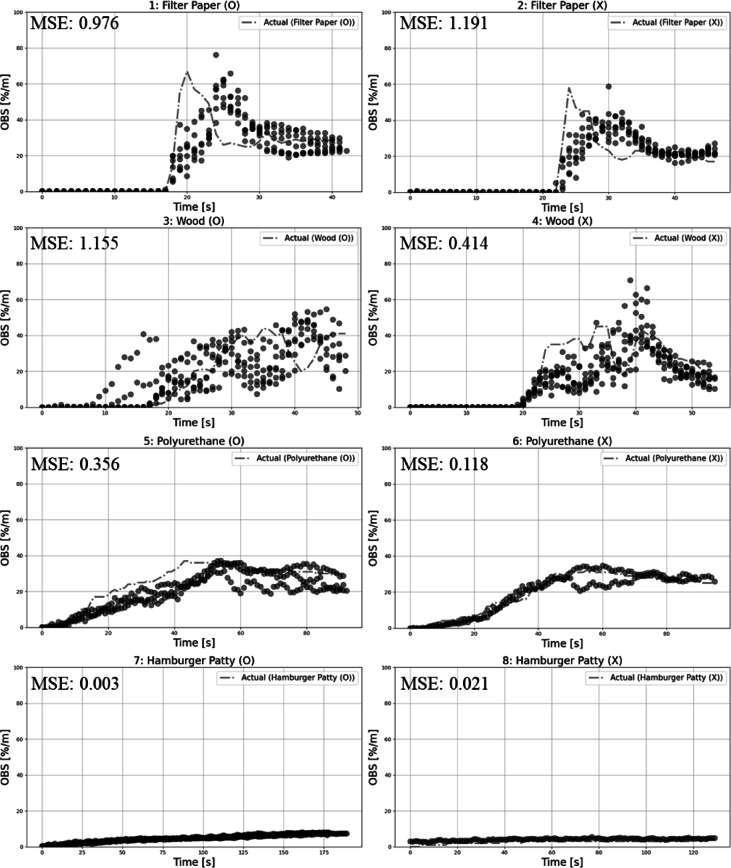
Regression results of CNN model.

The 940 nm region, classified as near-infrared (NIR), exhibits unique scattering and absorption properties compared to visible light due to interactions with smoke particles. For example, Fig. 11 illustrates the data collected during the paper fire test using the multi-wavelength smoke detector. The 940 nm scattering intensity (black solid line) shows significantly higher values compared to other wavelengths, highlighting its utility in distinguishing fire-related smoke from non-fire aerosols commonly encountered in indoor environments.

Recent studies have further explored the characteristics of the NIR wavelength range in smoke detection systems. For example, Li et al.^29^ developed a smoke detector incorporating the 940 nm wavelength to reduce false alarms. Their system classified aerosols, water, dust, and agglomerates, achieving improved false alarm detection accuracy without complex calculations. These findings align with the results of this study, underscoring the potential of NIR data to enhance fire detection system performance in challenging indoor environments.

The performance advantage of the MTL model over STL in smoke density prediction (MSE 0.668 vs. 1.858) can be attributed to the shared representation learned jointly across classification and regression tasks. In single-task learning, the regression model lacks access to discriminative features derived from source classification, resulting in a less informative feature space. In contrast, the shared encoder in the MTL framework acts as implicit regularization, improving generalization by simultaneously optimizing both tasks. This finding is consistent with the theoretical basis of multi-task learning proposed by Caruana^15^, who demonstrated that auxiliary tasks provide additional supervisory signals that enhance overall learning efficiency.

Among the evaluated architectures, the CNN-LSTM model achieved the best balance of classification accuracy (0.97) and regression performance (MSE 0.668). This can be explained by the complementary roles of its components: CNN layers extract local spectral patterns from multi-wavelength scattering signals, while LSTM layers capture the temporal evolution of smoke concentration over time. Since smoke generation is an inherently dynamic process, temporal dependencies in the scattering signals are critical for accurate density prediction. The relative underperformance of the Transformer model may be attributed to the short sequence length (time step = 30) used in this study, which limits the benefit of global self-attention mechanisms that typically excel with longer sequences.

Compared to existing multi-wavelength single-task approaches such as those by Özyurt^24,25^ and Li et al.^29^, which focus exclusively on aerosol source classification, the proposed MTL framework additionally estimates smoke density — a capability not addressed in prior work. Furthermore, while Baek et al.^27^ demonstrated the value of multi-sensor fusion for binary fire/non-fire detection, the present study extends this concept by enabling fine-grained source identification and quantitative hazard assessment within a single unified model, without the additional sensor hardware complexity.

Despite these promising results, several limitations must be acknowledged. The experimental dataset used in this study was aligned with the UL 268 standard, which includes representative fire sources such as filter paper, wood, polyurethane, and hamburger patties. While these sources account for a large proportion of residential and commercial fire incidents, they do not encompass the full diversity of combustible materials and aerosols encountered in real-world environments. Consequently, the observed prominence of the 940 nm wavelength may be partially influenced by the specific fire sources defined in the UL 268 framework.

It should also be acknowledged that the relatively small number of experiments per fire source (16 per category) may limit the statistical robustness of the reported results. While the sliding window approach and multi-sensor configuration substantially increase the effective training sample size, future work should incorporate a larger number of independent experimental repetitions to more rigorously assess model generalization across diverse ignition conditions and fire progression patterns.

At a more fundamental level, this limitation reflects an inherent challenge of fixed-label fire detection systems. In practical indoor environments, new materials, proprietary compounds, and process-specific substances are continuously introduced, often generating smoke characteristics that are not represented in standardized training datasets. Furthermore, real fire incidents frequently involve mixed-source combustion, where multiple materials ignite simultaneously or sequentially, producing complex smoke compositions with overlapping spectral signatures. For instance, in residential environments, wood furniture and synthetic foam may combust together, and simultaneous combustion of structural materials and process-specific compounds is common. Such scenarios generate smoke with composite scattering characteristics that may not correspond to any single source label within the current classification framework, potentially degrading model performance.

Beyond this, the experimental setup in this study was conducted under controlled conditions based on the UL 268 standard and does not fully account for environmental variations such as ventilation, ambient temperature, and humidity. These factors can influence the optical properties of smoke and may affect the generalization performance of the proposed model in real-world environments. Future work will address this limitation by incorporating diverse environmental conditions into the experimental design and developing compensation strategies to improve robustness under varying operational conditions.

Rather than treating these limitations as fixed constraints, the proposed framework is designed with adaptability in mind. The authors have previously investigated an LED array-based multi-angle light scattering approach^56^ for aspirating smoke detection, which incorporated temperature compensation strategies and minimized the influence of ambient airflow conditions. In addition, an adaptive progressive learning approach^57^ was developed to enable smoke detection models to incrementally incorporate previously unseen combustible materials with limited retraining data. Building on these efforts, future work will focus on constructing dedicated mixed-source experimental datasets—including combinations of UL 268 fire sources such as wood and polyurethane—incorporating diverse environmental conditions, and exploring multi-label classification frameworks to enable simultaneous identification of multiple contributing fire sources.

The proposed multi-task learning model supports early-stage fire detection by providing both qualitative fire source identification and quantitative smoke density estimation, enabling graduated emergency response strategies. Moreover, its low memory footprint (129.96 MB) and fast inference time (0.004 s) suggest its potential for deployment on resource-constrained edge devices within building fire detection and automation systems, pending validation on actual embedded hardware platforms. In practical terms, the simultaneous availability of fire source type and smoke density from a single lightweight model enables tiered alarm systems. For instance, early warnings can be issued at low OBS levels, while responses can be escalated as density increases. By integrating multi-wavelength sensing, multi-task learning, and adaptive re-learning mechanisms, the proposed approach establishes a foundation for fire detection systems that can maintain high reliability while evolving alongside changing operational conditions and minimizing false alarms in indoor environments. It should also be noted that although memory usage and inference time were analyzed to assess computational efficiency, the proposed system has not yet been fully validated on embedded hardware platforms. Future work will include real-world deployment and power efficiency evaluation on edge devices to further verify system reliability under practical constraints.

## Conclusions

This study aimed to address the limitations of traditional smoke detectors by proposing a novel smoke detection model capable of accurately classifying fire sources and predicting smoke density, enabling more effective fire detection and response. To achieve this, this study developed a multi-task learning model integrating fire source classification and smoke density prediction, utilizing data collected under various scenarios in compliance with the UL 268 standard. The main findings of this study are as follows:

1. The CNN-LSTM model demonstrated the best performance with an accuracy of 0.97 and an MSE of 0.668. It also recorded efficient results in terms of average inference time (0.004 s) and memory usage (129.91 MB). These findings validate the CNN-LSTM model’s ability to provide a balanced combination of performance and efficiency in a multi-task learning environment.
2. The Multi-task Learning (MTL) model reduced memory usage by approximately 45% and achieved inference times about 82% faster compared to Single-task Learning (STL) models. Additionally, the MTL model achieved lower prediction errors (MSE 0.668) for smoke density, confirming its suitability for real-time detection systems in dynamic environments.
3. The highest performance was achieved when all four wavelengths were used, with an observed trend of improved accuracy and predictive precision as the number of features increased. Notably, higher wavelengths, such as 660 nm and 940 nm, played a critical role in enhancing accuracy due to their pronounced scattering characteristics, enabling more reliable discrimination between fire smoke and non-fire aerosols, including dust and welding fumes.

This study demonstrates that a multi-task learning approach leveraging multi-wavelength data can significantly improve the performance of smoke detectors, providing valuable insights for their design and application in indoor environments. However, the current framework is limited to single-source fire scenarios, which may restrict its applicability in more complex real-world environments. To address this limitation, the authors have previously explored an adaptive progressive learning approach^57^ that incrementally incorporates new combustible materials with limited retraining data. Building on this, future work will further address this limitation by incorporating mixed-source combustion data and extending the model to better handle more diverse fire conditions.

By improving fire detection accuracy while reducing false alarms, the proposed model supports the development of safer and more efficient fire safety management systems in indoor environments. The system’s low memory footprint and fast inference time indicate its potential suitability for resource-constrained IoT-based fire detection networks. However, these characteristics were evaluated in a controlled laboratory setting, and actual deployment in industrial or building environments would require further validation on embedded hardware platforms under real operating conditions.

## Data Availability

The datasets generated and analysed during the current study are available from the corresponding author on reasonable request.

## Results of transformer model

See Fig. 12 and Table 10.

**Table 10:** Classification results of transformer model.

Confusion matrix				
	Precision	Recall	F1-score	Support
Normal	0.97	0.97	0.95	585
Paper	0.98	0.62	0.76	334
Wood	0.79	0.96	0.86	457
Polyurethane	0.99	0.95	0.97	441
Hamburger patty	0.94	0.99	0.97	479
Accuracy			0.92	2296
Macro avg	0.93	0.90	0.90	2296
Weighted avg	0.92	0.92	0.91	2296

## Results of CNN-GRU model

See Fig. 13 and Table 11.

**Table 11:** Classification results of CNN-GRU model.

Confusion matrix				
	Precision	Recall	F1-score	Support
Normal	0.99	0.97	0.98	585
Paper	0.95	0.94	0.94	334
Wood	0.95	0.97	0.96	457
Polyurethane	0.99	0.95	0.97	441
Hamburger patty	0.96	1.00	0.98	479
Accuracy			0.97	2296
Macro avg	0.97	0.97	0.97	2296
Weighted avg	0.97	0.97	0.97	2296

## Results of CNN model

See Fig. 14 and Table 12.

**Table 12:** Classification results of CNN model.

Confusion matrix				
	Precision	Recall	F1-score	Support
Normal	0.98	0.98	0.98	585
Paper	0.98	0.90	0.94	334
Wood	0.93	0.98	0.96	457
Polyurethane	0.99	0.97	0.98	441
Hamburger patty	0.97	1.00	0.98	479
Accuracy			0.97	2296
Macro avg	0.97	0.97	0.97	2296
Weighted avg	0.97	0.97	0.97	2296

## Abbreviations

MB: Megabyte
MSE: Mean squared error
CNN: Convolutional neural network
CNN-LSTM: Convolutional neural network-long short-term memory
CO: Carbon monoxide
CO_2_: Carbon dioxide
GRU: Gated recurrent unit
LED: Light emitting diode
LSTM: Long short-term memory
MTL: Multi-task learning
O_2_: Oxygen
OBS: Obscuration [%/m]
STL: Single-task learning
UL: Underwriters laboratories
